# Increase in Body Weight Following Residential Displacement: 5-year Follow-up After the 2011 Great East Japan Earthquake and Tsunami

**DOI:** 10.2188/jea.JE20190333

**Published:** 2021-05-05

**Authors:** Shuko Takahashi, Yuki Yonekura, Kozo Tanno, Haruki Shimoda, Kiyomi Sakata, Akira Ogawa, Seiichiro Kobayashi

**Affiliations:** 1 Division of Medical Education, Iwate Medical University, Iwate, Japan; 2 Takemi Program in International Health, Harvard T.H. Chan School of Public Health, Boston, MA, USA; 3 Department of Health and Welfare, Iwate Prefecture, Iwate, Japan; 4 St. Luke’s International University, Tokyo, Japan; 5 Department of Hygiene and Preventive Medicine, School of Medicine, Iwate Medical University, Iwate, Japan; 6 Iwate Medical University, Iwate, Japan

**Keywords:** earthquake, tsunami, natural disaster, body weight, social conditions

## Abstract

**Background:**

Previous studies have linked residential displacement as a result of the 2011 East Japan Earthquake to increases in body weight. However, no study has examined longer-term trajectories of body weight among displaced survivors. We compared body weight change between survivors relocated to temporary housing (TH) group versus other types of accommodation for up to 5 years after the Great East Japan Earthquake.

**Methods:**

Longitudinal follow-up was conducted from 2011 to 2015 in a cohort of 9,909 residents of 42,831. We compared trends in body weight in the TH group (*n* = 3,169) and the non-TH group (*n* = 6,740) using a mixed linear regression model stratified by sex (mean age, 61.0 years old; male, 38.9%).

**Results:**

In age-adjusted analysis, the body weight in the 2011 survey was not significantly different between two groups for either sex. In men, the TH group significantly increased body weight compared to the non-TH group since 2012. In women, body weight sharply increased in the TH group while body weight did not change in the non-TH group during survey time points. The interaction of living conditions and survey years was statistically significant in both sexes (men; F-value, 6.958; *P* < 0.001: women; F-value, 19.127; *P* < 0.001).

**Conclusion:**

Survivors relocated to temporary housing had an increased risk of weight gain. The weight gain in this group is a potential risk factor for metabolic syndrome in the post-disaster period.

## INTRODUCTION

The 2011 Great East Japan Earthquake and Tsunami resulted in widespread property destruction, and almost a quarter of million individuals ended up being displaced and relocated. Immediately after the disaster, people who lost their homes were evacuated to emergency shelters, such as public community centers.^1^ Several months later, they moved to prefabricated temporary housing (resembling the trailers used by the United States’ Federal Emergency Management Agency).

In a previous study, we showed that the people living in temporary housing experienced significantly increased body weight compared with people who managed to avoid moving to temporary housing (ie, they stayed in their own homes, or moved into rental housing on the private market). The estimated weight gain in the people living in the temporary housing was 0.52 kg (95% confidence interval [CI], 0.30–0.74) in men and 0.56 kg (95% CI, 0.42–0.71) in women than the people not living in temporary housing. However, our previous study involved relatively short follow-up, from 6 months to 24 months after the Great East Japan Earthquake.^2^ Even though the trailer homes were designated as “temporary” housing, in reality people ended up living in these shelters for 5 years after the disaster, while the government constructed more permanent housing.

Although other studies have documented increases in body weight during & after disaster, most of them involved short term observations.^3^^–^^8^ Only a few studies have examined the long-term trends in body weight after disaster.^9^^–^^12^ In particular, no study has evaluated the long-term trajectories of body weight according to different housing arrangements inside the tsunami-affected area.

## METHODS

### Study population

We used the data from the Research project for prospective Investigation of health problems Among Survivors of the Great East Japan Earthquake and Tsunami Disaster (RIAS) for analysis. The study is a longitudinal cohort study to determine the long-term health impact of the disaster. The study was started 6 months after the disaster and has been conducted every year in Yamada Town, Otsuchi Town, and Rikuzentakata City.^13^^–^^15^ The survivors were identified their addresses based on municipal government’s information. All residents aged 18 years or older were recruited by sending out notifications of the health survey and by announcing in a community bulletin board (total, 42,831). A total of 10,081 participants who provided written informed consent comprised the original cohort (23.5% of the total population). After excluding people those who lack data for at least one variable of sex, living condition and body weight of the 2011 (*n* = 172), we analyzed the participants regardless of their participation/non-participation during any given study wave between 2011 and 2015 (*n* = 9,909; mean age, 61.0 years; men, 38.9%).

### Measurements

#### Housing conditions

The respondents’ current housing situation was assessed via self-report.^2^ Based on the items in the 2011 and 2012 survey, we classified the living conditions into two groups: those living in temporary housing (TH) (prefabricated temporary housing and shelters) and those not living in temporary housing (non-TH).^2^ In the survey from 2013 to 2015, we asked participants “Currently, where do you live mostly?”, with responses classified into two groups: a temporary housing (TH) group (prefabricated temporary housing) and a not temporary housing (non-TH) group (same house as that during the disaster; post-disaster public-funded rental accommodation; relocated to a rental apartment except emergency provisional housings by making use of privately-rented housings; rebuilding a house on the same place as the disaster; new house built in different place from that before the disaster; family’s, friend’s, or relative’s house; and others).

#### Variables

Variables were measured at each time point from 2011 to 2015. Body weight (kg) and height were measured using digital scales without shoes. Body mass index (BMI) was calculated as body weight (kg)/height (m^2^). We measured blood pressure and blood tests associated with body weight change including systolic blood pressure (SBP; mm Hg), diastolic blood pressure (DBP; mm Hg), total cholesterol (TC; mg/dL), high-density lipoprotein cholesterol (HDLC; mg/dL), triglyceride (TG; mg/dL) and glycosylated hemoglobin (HbA1c; %). Fasting or non-fasting blood samples were drawn from the antecubital veins. Self-report questionnaires were administered to assess age, lifestyle (smoking status, alcohol drinking status, daily physical activity, the average number of meals per day and dietary intake), socioeconomic status (economic status), psychosocial factors (psychological distress and insomnia), and social factors (social network and social capital) of participants after the disaster. Based on the previous study, physical activity was classified into two categories: low physical activity (<23 METs per week) and normal physical activity (≥23 METs per week). The average number of meals per day during the past several days was dichotomized the responds into a small number of meals (<3 times a day) versus a normal number of meals (≥3 times a day). Dietary intake dichotomized into good dietary intake or poor dietary intake based on the previous study.^16^ Economic status was categorized into two groups: severe economic status or usual. Psychological distress dichotomized into those with psychological distress (scores of 5–24) and those with no psychological distress (score of 0–4) using the K6 scale in Japan.^17^^,^^18^ Insomnia was classified as those with insomnia (scores of 6–24) and those with no insomnia (scores of 0–5) on the Athens Insomnia Scale (AIS).^19^^–^^22^ Social network was assessed by the Lubben’s Social Network Scale.^23^^,^^24^ Social capital was evaluated using four questions regarding social cohesion about residents’ perceptions of trust in the community and levels of mutual help.^16^ We used a cut-off point of ≤9 and dichotomized responses into low level and high level.^25^ Obesity was identified as BMI of ≥25 kg/m^2^. Participants were asked about past medical history including the status of prescribed drugs for hypertension, dyslipidemia, and diabetes mellitus previously described in detail.^2^

### Statistical analysis

We compared baseline characteristics in the 2011’s survey including age, body weight, BMI, SBP, DBP, TC, HDLC, TG, HbA1c, smoking status, alcohol drinking status, physical activity, average number of meals per day, dietary intake, economic status, psychological distress, insomnia, social network, social capital, overweight, hypertension, dyslipidemia and diabetes mellitus between living conditions in 2011. In the crude analysis, the Student’s *t*-test was used for continuous variables and the Chi-squared test was used for categorical variables. In the age adjusted analysis, analysis of covariance was used for continuous value and logistic regression analysis was used for categorical values.

We analyzed body weight as an independent variable from the five time points (2011, 2012, 2013, 2014, and 2015) using linear mixed effect models, stratified by sex. We included explanatory variables of age, time, living conditions, the interaction between living conditions and time points as fixed effects, and we included the individual as a random effect. We performed the main analysis without the one wave participants (*n* = 1,592) considering the change of body weight (*n* = 8,319). The coefficients of body weight in the fixed effects were calculated introducing a range of confounding variables in the two models: model 1 included age, time, living conditions, the interaction between living conditions and time points; model 2 included the variables from model 1 with additional adjustment for smoking status, alcohol drinking status, physical activity, the number of meals (<3 times), dietary intake, economic status, psychological distress, insomnia, social network and social capital. The equation used in the regression model was as below: Body weight = β0 + β1 × TH (TH/non-TH) + β2 × 2012 + β3 × 2013 + β4 × 2014 + β5 × 2015 + β6 × 2012 × TH (TH/non-TH) + β7 × 2013 × TH (TH/non-TH) + β8 × 2014 × TH (TH/non-TH) + β9 × 2015 × TH (TH/non-TH) + β10 × age in Model 1. In Model 2, body weight = β0 + β1 × TH (TH/non-TH) + β2 × 2012 + β3 × 2013 + β4 × 2014 + β5 × 2015 + β6 × 2012 × TH (TH/non-TH) + β7 × 2013 × TH (TH/non-TH) + β8 × 2014 × TH (TH/non-TH) + β9 × 2015 × TH (TH/non-TH) + β10 × age + β11 × current smokers (current smokers/non-current smokers) + β12 × drinkers (drinkers/non-drinkers) + β13 × low physical activity (low physical activity/normal physical activity + β14 × small number of meals (small number of meals/normal number of meals) + β15 × poor dietary intake (poor dietary intake/good dietary intake) + β16 × severe economic status (severe economic status/usual economic status) + β17 × psychological distress (psychological distress/no psychological distress) + β18 × insomnia (insomnia/no insomnia) + β19 × low level of social network (low level of social network/high level of social network) + β20 × low level of social capital (low level of social capital/high level of social capital). We showed an estimated marginal mean of body weight in living conditions in each survey point. Next, we performed the same analysis with the first wave participants (*n* = 9,909), and conducted the following analyses in these subjects. We also conducted analysis with the missing covariate data in 2011 by being imputed in multiple imputation using Markov Chain Monte Carlo method by creating five imputed datasets. We combined the results from each imputed dataset to obtain the final estimates. We combined the results from each imputed dataset to obtain the final estimates. In subgroup analysis, we demonstrated the coefficients of body weight in the interaction effect between living conditions and time points stratified by obesity in the 2011 survey.

All *P*-values were based on two-sided tests, and *P*-values <0.05 were considered statistically significant. The Statistical Package for Social Sciences version 24.0 (IBM Corp, Armonk, NY, USA) was used for all analyses. The research plan was approved by the Ethics Committee of Iwate Medical University (approval no. H23-69).

## RESULTS

Table 1 shows the baseline characteristics. The number of participants was 3,169 in the TH group, and 6,740 in the non-TH group. The number of participants in the TH group gradually decreased over the survey years, considering that participants moved to non-temporary housing, such as a rental apartment or a new house (percentages in 2015: the TH group, approximately 19%; the non-TH group, 80%) (eTable 1). Men in the TH group were significantly younger compared to the non-TH group. Ages for women were similar in the two groups. Although the people in the TH group had significantly higher body weight compared with those in the non-TH group in men in crude analysis that difference disappeared in age-adjusted analysis. In women, there was not a significant difference of body weight between the two groups in either crude or age-adjusted analysis.

**Table 1.** Baseline characteristics of participants in the 2011’s survey (*n* = 9,909)

Men (*n* = 3,852)		Missing	TH (*n* = 1,231)	non-TH (*n* = 2,621)	*P* Value	TH (*n* = 1,231)	non-TH (*n* = 2,621)	*P* Value
		
		*n* (%)	Mean (SD)/*n* (%)	Mean (SD)/*n* (%)		Adjusted mean (SE)/Adjusted proportion (SE)	Adjusted mean (SE)/Adjusted proportion (SE)	
Age	Age, years	0 (0.0)	61.4 (14.2)	62.8 (14.4)	0.004			
Anthropometrical examination	Body weight, kg	0 (0.0)	66.4 (11.1)	65.4 (10.6)	0.007	66.2 (0.3)	65.5 (0.2)	0.068
	BMI, kg/m^2^	0 (0.0)	24.4 (3.4)	24.2 (3.2)	0.152	24.3 (0.1)	24.2 (0.1)	0.254
Blood pressure	SBP, mm Hg	1 (0.0)	128.2 (17.3)	129.5 (17.8)	0.031	128.4 (0.5)	129.4 (0.3)	0.096
	DBP, mm Hg	1 (0.0)	77.1 (10.7)	76.9 (11.1)	0.498	77.1 (0.3)	76.9 (0.2)	0.496
Blood tests	TC, mg/dL	0 (0.0)	199.4 (34.7)	197.5 (35.3)	0.114	199.0 (1.0)	197.6 (0.7)	0.239
	HDLC, mg/dL	0 (0.0)	59.2 (16.8)	59.1 (16.8)	0.880	59.2 (0.5)	59.1 (0.3)	0.860
	TG, mg/dL	0 (0.0)	163.5 (120.6)	152.2 (100.9)	0.002	162.3 (3.0)	152.8 (2.1)	0.010
	HbA1c, %	0 (0.0)	5.73 (0.72)	5.75 (0.78)	0.362	5.74 (0.02)	5.75 (0.02)	0.637
Life style	Current smokers	0 (0.0)	426 (34.6)	769 (29.3)	0.001	32.6 (1.4)	28.4 (0.9)	0.009
	Drinkers	0 (0.0)	781 (63.4)	1,603 (61.2)	0.173	63.3 (1.4)	61.2 (1.0)	0.208
	Low physical activity	18 (0.5)	793 (64.7)	1,543 (59.2)	0.001	65.0 (1.4)	59.1 (1.0)	0.001
	Small number of meals (<3 times)	28 (0.7)	120 (9.9)	170 (6.5)	<0.001	7.6 (1.0)	5.2 (0.7)	<0.001
	Poor dietary intake	0 (0.0)	574 (46.6)	1,083 (41.3)	0.002	46.0 (1.4)	41.4 (1.0)	0.008
Socioeconomic status	Severe economic status	14 (0.4)	783 (64.0)	1,269 (48.5)	<0.001	64.0 (1.4)	48.9 (1.0)	<0.001
Psychological factors	Psychological distress	36 (0.9)	496 (40.7)	878 (33.8)	<0.001	40.2 (1.4)	33.8 (0.9)	<0.001
	Insomnia	39 (1.0)	379 (31.1)	593 (22.9)	<0.001	30.9 (1.3)	22.8 (0.8)	<0.001
Social factors	Low level of social network	75 (1.9)	530 (43.9)	1,085 (42.2)	0.327	43.7 (1.4)	42.3 (1.0)	0.426
	Low level of social capital	8 (0.2)	143 (11.6)	290 (11.1)	0.609	11.5 (0.9)	11.1 (0.6)	0.700
Cardiovascular risk factors	Obesity	0 (0.0)	462 (37.5)	984 (37.5)	0.994	37.3 (1.4)	37.6 (0.9)	0.866
	Hypertension	1 (0.0)	605 (49.1)	1,332 (50.8)	0.333	49.8 (1.5)	49.9 (1.0)	0.980
	Dyslipidemia	0 (0.0)	433 (35.2)	881 (33.6)	0.340	34.9 (1.4)	33.6 (0.9)	0.457
	Diabetes mellitus	0 (0.0)	174 (14.1)	372 (14.2)	0.961	14.0 (1.0)	13.0 (0.7)	0.696

Women (*n* = 6,057)		Missing	TH (*n* = 1,938)	non-TH (*n* = 4,119)	*P* Value	TH (*n* = 1,938)	non-TH (*n* = 4,119)	*P* Value
		
		*n* (%)	Mean (SD)/*n* (%)	Mean (SD)/*n* (%)		Adjusted mean (SE)/Adjusted proportion (SE)	Adjusted mean (SE)/Adjusted proportion (SE)	
Age	Age, years	0 (0.0)	59.7 (15.2)	60.5 (14.3)	0.051			
Anthropometrical examination	Body weight, kg	0 (0.0)	54.1 (9.6)	53.9 (9.0)	0.483	54.0 (0.2)	53.9 (0.1)	0.722
	BMI, kg/m^2^	12 (0.2)	23.4 (3.7)	23.4 (3.7)	0.717	23.4 (0.1)	23.4 (0.1)	0.890
Blood pressure	SBP, mm Hg	0 (0.0)	123.2 (18.6)	124.9 (19.5)	0.002	123.5 (0.4)	124.7 (0.3)	0.012
	DBP, mm Hg	0 (0.0)	72.1 (10.8)	72.7 (10.7)	0.026	72.2 (0.2)	72.7 (0.2)	0.067
Blood tests	TC, mg/dL	1 (0.0)	209.4 (35.5)	209.0 (35.8)	0.658	209.5 (0.8)	208.9 (0.6)	0.517
	HDLC, mg/dL	1 (0.0)	67.4 (16.7)	66.6 (16.4)	0.093	67.3 (0.4)	66.7 (0.3)	0.168
	TG, mg/dL	1 (0.0)	125.5 (75.4)	129.9 (77.7)	0.039	125.9 (1.7)	129.7 (1.2)	0.066
	HbA1c, %	1 (0.0)	5.60 (0.51)	5.64 (0.60)	0.011	5.61 (0.01)	5.64 (0.01)	0.034
Life style	Current smokers	0 (0.0)	159 (8.2)	273 (6.6)	0.026	5.2 (0.5)	4.5 (0.3)	0.163
	Drinkers	0 (0.0)	314 (16.2)	572 (13.9)	0.017	13.9 (0.8)	12.3 (0.5)	0.079
	Low physical activity	43 (0.7)	1,382 (71.6)	2,714 (66.5)	<0.001	71.8 (1.0)	66.5 (0.7)	0.000
	Small number of meals (<3 times)	37 (0.6)	123 (6.4)	206 (5.0)	0.028	4.3 (0.4)	3.6 (0.3)	0.134
	Poor dietary intake	0 (0.0)	668 (34.5)	1,274 (30.9)	0.006	33.8 (1.1)	30.7 (0.7)	0.014
Socioeconomic status	Severe economic status	15 (0.2)	1,148 (59.4)	1,926 (46.9)	<0.001	59.4 (1.1)	47.0 (0.8)	<0.001
Psychological factors	Psychological distress	123 (2.0)	1,003 (53.1)	1,815 (44.9)	<0.001	53.0 (1.1)	44.9 (0.8)	<0.001
	Insomnia	99 (1.6)	847 (44.4)	1,425 (35.2)	<0.001	44.4 (1.1)	35.1 (0.8)	<0.001
Social factors	Low level of social network	127 (2.1)	769 (40.9)	1,637 (40.4)	0.724	40.7 (1.1)	40.4 (0.8)	0.825
	Low level of social capital	23 (0.4)	177 (9.2)	347 (8.5)	0.366	9.1 (0.7)	8.5 (0.4)	0.378
Cardiovascular risk factors	Obesity	12 (0.2)	574 (29.7)	1,218 (29.6)	0.982	29.7 (1.0)	29.4 (0.7)	0.859
	Hypertension	0 (0.0)	822 (42.4)	1,733 (42.1)	0.802	39.6 (1.3)	38.0 (0.9)	0.301
	Dyslipidemia	1 (0.0)	906 (46.7)	1,860 (45.2)	0.246	46.9 (1.1)	44.9 (0.8)	0.146
	Diabetes mellitus	2 (0.0)	144 (7.4)	292 (7.1)	0.632	6.5 (0.6)	6.1 (0.4)	0.526

DBP, diastolic blood pressure; HbA1c, glycosylated hemoglobin; HDLC, high-density lipoprotein cholesterol; SBP, systolic blood pressure; SD, standard deviation; SE, standard error; TC, total cholesterol; TH, temporary housing group.

In the crude analysis, continuous variables indicate the mean (standard deviation), categorical variables indicate the number of case (percentage). *P* Values were calculated using Student’s *t*-tests for continuous variables and the Chi square test for categorical variables.

In the age-adjusted analysis, the continuous variables indicate adjusted mean (standard error) and the categorical variables indicate adjusted proportion (standard error). Means and standard deviations were calculated using analysis of covariance for continuous values, and the number of cases and percentages were calculated using logistic regression analysis for categorical values.

**Figure 1.**
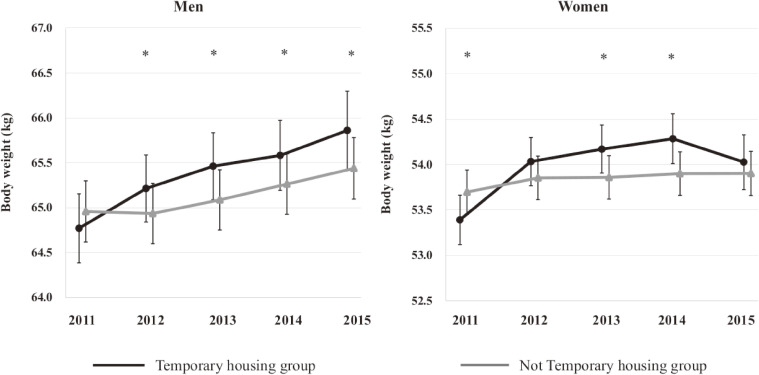
The trend of estimated marginal mean of body weight

Table 2 shows the coefficients of mixed effects during survey time points using liner mixed effect models (*n* = 8,317). There was a significant difference in the effect of time between living conditions in both models (“2012 × TH,” “2013 × TH,” “2014 × TH,” and “2015 × TH,” *P* Value <0.05). We further performed the same analysis with the first wave participants in two models (*n* = 9,909) (eTable 2). The results were similar in both models as our main results in both sexes. In the analysis with missing covariate data in the 2011 using multiple imputation methods, the difference in the effect of time between living conditions was significant in each point in both sexes (eTable 3). Figure 1 shows the trend of estimated marginal mean of body weight from 2011 to 2015. In men, although there was no significant difference in body weight in 2011 between the two groups; the TH group had significantly higher body weight than the non-TH group in 2012. Body weight in the TH group was significantly higher than in the non-TH group from 2012 to 2015 (eTable 1 and eTable 4). In women, although body weight in the TH group was lower than that in the non-TH group at baseline (in 2011), body weight significantly increased in the TH group while body weight did not change in the non-TH group during the survey time points. Body weight in the TH group was significantly higher than that in the non-TH group from 2013 to 2014. The significant difference was attenuated in 2015. The interaction of living conditions and time was statistically significant in both sexes (men; F-value, 6.958; *P* < 0.001: females; F-value, 19.127; *P* < 0.001). The coefficients of interactions stratified by obesity in the 2011 survey were statistically significant in all items (eTable 5). In the obese group there was a non-significant interaction effect between body weight × living conditions compared to the non-obese group in both sexes (eTable 6).

**Table 2.** Coefficient of mixed effects using linear mixed effects models

	Men						Women					
	
	Model 1 (*n* = 3,145)			Model 2 (*n* = 3,142)			Model 1 (*n* = 5,172)			Model 2 (*n* = 5,170)		
	Coefficient (kg)	95% CI		Coefficient (kg)	95% CI		Coefficient (kg)	95% CI		Coefficient (kg)	95% CI	
TH	−0.21	−0.50 to 0.09		−0.14	−0.44 to 0.16		−0.35	−0.55 to −0.14	^*^	−0.32	−0.53 to −0.11	^*^
2011	Base			Base			Base			Base		
2012	−0.02	−0.13 to 0.09		0.02	−0.09 to 0.14		0.15	0.08 to 0.23	^*^	0.18	0.10 to 0.26	^*^
2013	0.14	−0.01 to 0.29		0.16	0.00 to 0.32		0.16	0.05 to 0.26	^*^	0.20	0.09 to 0.31	^*^
2014	0.32	0.13 to 0.51	^*^	0.39	0.19 to 0.59	^*^	0.20	0.07 to 0.33	^*^	0.25	0.12 to 0.39	^*^
2015	0.50	0.28 to 0.72	^*^	0.59	0.35 to 0.82	^*^	0.20	0.05 to 0.35	^*^	0.27	0.11 to 0.44	^*^
2012 × TH	0.47	0.29 to 0.65	^*^	0.44	0.25 to 0.63	^*^	0.50	0.37 to 0.62	^*^	0.46	0.32 to 0.59	^*^
2013 × TH	0.57	0.31 to 0.83	^*^	0.59	0.33 to 0.86	^*^	0.64	0.46 to 0.81	^*^	0.63	0.45 to 0.81	^*^
2014 × TH	0.52	0.21 to 0.84	^*^	0.45	0.12 to 0.77	^*^	0.72	0.51 to 0.93	^*^	0.69	0.47 to 0.91	^*^
2015 × TH	0.63	0.24 to 1.01	^*^	0.55	0.15 to 0.94	^*^	0.46	0.20 to 0.72	^*^	0.46	0.19 to 0.72	^*^
Age	−0.28	−0.30 to −0.25	^*^	−0.29	−0.31 to −0.26	^*^	−0.13	−0.14 to −0.11	^*^	−0.13	−0.15 to −0.11	^*^
Current smoking				−1.16	−1.45 to −0.87	^*^				−0.83	−1.20 to −0.47	^*^
Alcohol drinking				0.48	0.30 to 0.65	^*^				0.20	0.05 to 0.35	^*^
Low physical activity				0.12	0.02 to 0.21	^*^				0.09	0.02 to 0.15	^*^
Small number of meals (<3 times)				0.27	0.04 to 0.51	^*^				−0.41	−0.59 to −0.23	^*^
Poor dietary intake				−0.03	−0.12 to 0.06					−0.05	−0.11 to 0.02	
Severe economic status				−0.02	−0.13 to 0.08					−0.02	−0.09 to 0.05	
Psychological distress				−0.07	−0.19 to 0.04					−0.10	−0.17 to −0.03	^*^
Insomnia				−0.02	−0.15 to 0.11					−0.06	−0.14 to 0.01	
Low level of social network				−0.04	−0.13 to 0.06					0.01	−0.06 to 0.08	
Low level of social capital				0.03	−0.10 to 0.17					−0.03	−0.13 to 0.07	

CI, confidence interval; TH group, temporary housing group.

^*^Statistically significant (*P* Values <0.05).

Model 1: adjustment for age, time, living conditions, the interaction between living conditions and time points.

Model 2: model 1 plus smoking status, alcohol drinking status, physical activity, the number of meals (<3 times), dietary intake, economic status, psychological distress, insomnia, social network and social capital.

Coefficients and 95% confidence were calculated using the linear mixed effect models.

## DISCISSION

We found the people in TH group experienced significant increases in body weight compared with the people in non-TH group in both sexes during 5 years of follow-up after the Great East Japan Earthquake. The significant differences remained after the adjustment for several confounding factors. Living in temporary housing was independently associated with weight gain for up to 5 years after the Great East Japan Earthquake, irrespective of baseline obesity.

Our study areas were not affected by the accident of Nuclear Power Plant in Fukushima (about 200 km away). A major strength of our study is the long term observation—up to 5 years after the disaster. Previous studies have suggested change of body weight after a natural disaster.^3^^–^^12^ Body weight in some of these studies were examined a short time after the disaster (within 2 years). A few studies investigated body weight for longer periods after the disaster.^9^^–^^12^ In Italy, Trevisan et al and Bland et al examined coronary heart disease risk factors among Italian male factory workers 7 years after the Irpinia earthquake. While the subjects exposed to the earthquake significantly increased body weight compared with those with non-exposed to the earthquake 2 months after the disaster,^5^ that difference had disappeared 7 years after the earthquake. Although studies were consistent with our study in terms of follow-up periods after the disaster (a long time after the disaster), the subjects in those studies were only male factory workers. In Japan, two studies in Fukushima compared healthcare data across the Great East Japan Earthquake for a long time.^11^^,^^12^ Ebner et al showed that body weight of Kawauchi village citizens increased in 2012 and 2013.^12^ Nomura et al revealed evacuees had significantly higher BMI compared with non-evacuees/temporary-evacuees from 2012 to 2014.^11^ Those studies were incompatible with our study in terms of the subjects; our subjects experienced living conditions inside tsunami-affected areas instead of evaluating subjects outside of the affected area. In addition, because Fukushima was affected by the Fukushima Diichi Nuclear Power Plant accident, there might be unique circumstances in that area (eg, people were forced to evacuate outside restricted area designated by the government to avoid high level radiation exposure). To the best of our knowledge, this is the first study to compare body weight by living conditions with a long follow-up after a natural disaster. Our study developed new findings regarding long-term health impacts after a natural disaster in community-dwelling people in a tsunami-affected area.

Differences in body weight between men and women were observed. After the 2011 survey when participants in the TH group had increased body weight than those in the non-TH group, men’s body weight steadily increased both in the TH and non-TH groups. In women, the TH group had a peak point in 2014 and subsequently decreased in 2015, while the non-TH group had almost similar body weight throughout the study period. Although we could not pinpoint the exact reason for body weight attenuated in women in the TH group, there are some possible explanations. First, preventive practices to avoid weight gain might be a significant factor for women in 2015. Following the disaster, several healthcare workers and non-governmental organizations (NGOs) sought to support survivors to participate in healthcare classes, particularly for individuals living in TH.^26^ Because most of the participants were women in these healthcare classes,^27^^,^^28^ women improved their lifestyle, such as improvement in their physical activity or following an appropriate dietary regimen, consequently reducing women’s body weight in the 5 years after the disaster. Second, it is assumed that the decreased body weight among women in the TH group is possibly due to chance. If we measure the body weight from 2016 onward, this phenomenon might be further explained. Finally, it appeared that men and women have different levels of susceptibility, with distinct vulnerable periods influenced by environmental change after natural disasters. It is notable that sex differences as a result of health and illness are associated with a number of determinants.^29^

Though the reasons underlying the association between body weight gain and housing conditions have not been identified in this study, there are some possible explanations. First, increased body weight was associated with changes in nutrition. People who evacuated to emergency shelters initially experienced shortages in specific foods, such as fresh vegetables, meat, and fish.^30^ We further analyzed the detailed information of dietary intakes (eTable 7). The participants in the TH group had significantly lower frequency of intakes of fish and shellfish, egg, soybean and related products, and vegetables compared with those in the non-TH group in both sexes (*P* < 0.05), a result consistent with the results of previous studies stating that survivors had short supply of fresh foods (vegetables and fish)^1^ and that individuals in difficult living conditions after the disaster were likely to have a lower prudent dietary pattern (high intake of vegetables, fruits, seafood, and soy foods).^31^ Dietary pattern, including prudent dietary pattern, is associated with body weight changes.^32^^–^^34^ By contrast, preserved foods were more available, including large quantities of processed food, which were voluntarily donated to evacuees from other non-affected regions, compared to non-preserved foods.^1^ These foods, with high calorie count from sugar and fats, may have contributed to an increase in body weight in the early phase after the disaster.^35^ Second, relocation to temporary shelters may have been associated with a change in the local food environment. For example, Hikichi et al reported that in Iwanuma (a tsunami-affected city in the neighboring Miyagi Prefecture), survivors who were relocated to temporary housing settlements ended up (inadvertently) moving closer to fast food outlets and bars.^36^ By “improving” access to unhealthy sources of food, the disaster recovery process may have unintentionally contributed to unhealthy weight gain. Third, the environmental changes as a result of disaster might be associated with increased body weight. A major factor causing body weight gain is energy imbalance, which is associated with the individuals’ environmental conditions and behaviors. Individuals are subsequently moving to temporary housing, which is mostly built in an inconvenient area such as mountainous spots. Families living in uncomfortable housing with narrow spaces will likely experience psychological distress and insomnia, a finding that was consistent with our findings (Table 1). Psychological problems, such as depression, are associated with difficulty with self-care,^37^ and short sleep duration is associated with obesity according to a meta-analysis.^38^ Furthermore, Goryoda et al have reported that low level of social capital after the Great East Japan Earthquake was associated with poor dietary intake.^16^ Yoshimura et al revealed that individuals living in TH had decreased physical activity.^39^ Moreover, environmental changes in the individuals’ living condition might result in a sedentary lifestyle. With psychological distress, insomnia, and low physical activity, we speculate that individuals will experience increased body weight 5 years after the disaster. Fourth, people in TH group might be from more disadvantaged socioeconomic circumstances compared with the non-TH group in tsunami affected area. In the Great East Japan Earthquake, the tsunami struck an area of Japan that was historically known to be in the risk zone for events of this type.^40^ Some residents built their homes on tsunami flood-risk areas because of limited land supply along a deeply indented coastline. Such people were more likely to be economically disadvantaged compared to those who settled in more secure areas.^36^ In present study, the people living in TH group also had higher percentage of economic difficulty in baseline characteristics. Because low socioeconomic status is associated with body weight gain,^41^ people in the TH group in a low socioeconomic state might have already been on a trajectory to increased body weight, irrespective of the experience of living in the TH group.

The present study had several limitations. First, there might be residual variables related to body weight change, such as socioeconomic status, amount of intake food, dietary patterns, and accurate physical activity, which could not be included. We could not determine the precise mechanisms from living conditions to body weight gain. Second, the generalizability of the present results is limited because the participants in the survey were relatively higher age with regard to population census.^13^ Because the people participated in this study might be high health consciousness and better access to health care facilities than those not participated in it, our results might be underestimated. But we believe those results were true phenomenon in a health impact in a large-scale natural disaster.

In conclusion, we have shown the people in the TH group significantly increased body weight than the people in the NTH group in 5 years following the Great East Japan Earthquake in men and women. Because an increase of body weight is an independent risk factor of cardiovascular disease, increase incidence of cardiovascular disease is a concern among survivors in the post-disaster period. In a future catastrophic natural disaster, we should target intervention at the people living in temporary housing appropriately to control body weight in order to prevent disaster survivors from suffering obesity-related diseases.

## APPENDIX A. SUPPLEMENTARY DATA

eTable 1. Comparison of body weight at each time point from 2011 to 2015 between living conditions

eTable 2. Coefficient of body weight in mixed effects using linear mixed effects models with the one wave participants

eTable 3. Coefficient of body weight in mixed effects using linear mixed effects models with the missing covariate data in the 2011 survey by multiple imputation methods

eTable 4. Pairwise comparison of the difference of body weight in living conditions using linear mixed effect models

eTable 5. The coefficients for body weight between time and living conditions stratified by obesity in the 2011 survey using multivariate linear mixed models

eTable 6. Coefficient of mixed effects with the interaction in time, living conditions, and obesity in the 2011 survey using linear mixed effects models

eTable 7. Detailed characteristics of the participants in the 2011 survey (n = 9,909)
