# Supplementary material for: Increase in Body Weight Following Residential Displacement: 5-year Follow-up After the 2011 Great East Japan Earthquake and Tsunami
Source: J Epidemiol. 2021 May 5;31(5):328–34. doi: 10.2188/jea.JE20190333 (PMC8021881; doi:10.2188/jea.JE20190333)
Supplement: Supplementary file 1 [file je-31-328-s001.pdf]

eTable 1. Comparison of body weight at each time point from 2011 to 2015 between living conditions

Men		Number of cases				Crude analysis			Age-adjusted analysis		
		Participants	Missing	TH	non-TH	TH (n=1,231)	non-TH (n=2,621)		TH (n=1,231)	non-TH (n=2,621)	
		n (%)	n (%)	n (%)	n (%)	Mean (SD)	Mean (SD)	*P* Value	Adjusted mean (SE)	Adjusted mean (SE)	*P* Value
**Body weight, kg**	**2011**	3,852 (100.0)	0 (0.0)	1,231 (32.0)	2,621 (68.0)	66.4 (11.1)	65.4 (10.6)	0.007 *	66.2 (0.3)	65.5 (0.2)	0.068
	**2012**	2,703 (70.2)	1,149 (29.8)	848 (31.4)	1,855 (68.6)	66.5 (11.5)	64.8 (10.3)	<0.001 *	66.1 (0.3)	65.0 (0.2)	0.005 *
	**2013**	2,527 (65.6)	1,325 (34.4)	687 (27.2)	1,840 (72.8)	66.4 (11.5)	64.6 (10.0)	<0.001 *	65.9 (0.4)	64.8 (0.2)	0.010 *
	**2014**	2,442 (63.4)	1,410 (36.6)	565 (23.1)	1,877 (76.9)	66.2 (11.6)	64.4 (9.9)	<0.001 *	65.6 (0.4)	64.6 (0.2)	0.044 *
	**2015**	2,348 (61.0)	1,504 (39.0)	450 (19.2)	1,898 (80.8)	67.1 (12.2)	64.5 (10.1)	<0.001 *	66.4 (0.47)	64.7 (0.23)	0.001 *

Women		Number of cases				Crude analysis			Age-adjusted analysis		
		Participants	Missing	TH	non-TH	TH (n=1,938)	non-TH (n=4,119)		TH (n=1,938)	non-TH (n=4,119)	
		n (%)	n (%)	n (%)	n (%)	Mean (SD)	Mean (SD)	*P* Value	Adjusted mean (SE)	Adjusted mean (SE)	*P* Value
**Body weight, kg**	**2011**	6,057 (100.0)	0 (0.0)	1,938 (32.0)	4,119 (68.0)	54.1 (9.6)	53.9 (9.0)	0.483	54.0 (0.2)	53.9 (0.1)	0.722
	**2012**	4,535 (74.9)	1,522 (25.1)	1,399 (30.8)	3,136 (69.2)	54.4 (9.8)	53.8 (8.9)	0.036 *	54.4 (0.2)	53.8 (0.2)	0.046 *
	**2013**	4,278 (70.6)	1,779 (29.4)	1,151 (26.9)	3,127 (73.1)	54.3 (9.6)	53.8 (9.1)	0.116	54.3 (0.3)	53.9 (0.2)	0.155
	**2014**	4,062 (67.1)	1,995 (32.9)	944 (23.2)	3,118 (76.8)	54.5 (9.6)	53.7 (9.2)	0.03 *	54.5 (0.3)	53.8 (0.2)	0.036 *
	**2015**	4,019 (66.4)	2,038 (33.6)	779 (19.4)	3,240 (80.6)	54.2 (9.5)	53.8 (9.2)	0.271	54.1 (0.3)	53.8 (0.2)	0.295

TH, temporary housing group; SD, standard deviation; SE, standard error.
Body weight indicates the mean (standard deviation) in the crude analysis and adjusted mean (standard error) in the age-adjusted analysis.
Means and standard deviations were calculated using Student's t-tests in the crude analysis and using analysis of covariance in the age-adjusted analysis.
*Statistically significant (*P* Value<0.05).

eTable 2. Coefficient of body weight in mixed effects using linear mixed effects models with the one wave participants

	Men				Women			
	Model 1 (n=3,852)		Model 2 (n=3,818)		Model 1 (n=6,057)		Model 2 (n=6,004)	
	Coefficient (kg)	95% CI	Coefficient (kg)	95% CI	Coefficient (kg)	95% CI	Coefficient (kg)	95% CI
**TH**	-0.19	-0.48 to 0.10	-0.12	-0.42 to 0.18	-0.31	-0.51 to -0.10	-0.28	-0.49 to -0.07
**2011**	Base		Base		Base		Base	
**2012**	-0.02	-0.13 to 0.08	0.02	-0.10 to 0.13	0.16	0.08 to 0.23	0.19	0.11 to 0.27
**2013**	0.13	-0.02 to 0.28	0.15	-0.01 to 0.31	0.16	0.06 to 0.26	0.21	0.09 to 0.32
**2014**	0.30	0.12 to 0.49	0.37	0.18 to 0.57	0.20	0.08 to 0.33	0.26	0.13 to 0.40
**2015**	0.48	0.26 to 0.70	0.56	0.33 to 0.79	0.21	0.06 to 0.35	0.28	0.12 to 0.44
**2012 × TH**	0.46	0.28 to 0.65	0.43	0.24 to 0.62	0.49	0.36 to 0.61	0.44	0.31 to 0.58
**2013 × TH**	0.56	0.30 to 0.82	0.58	0.31 to 0.84	0.62	0.44 to 0.79	0.61	0.43 to 0.79
**2014 × TH**	0.51	0.19 to 0.82	0.43	0.11 to 0.76	0.69	0.48 to 0.90	0.66	0.44 to 0.88
**2015 × TH**	0.61	0.23 to 1.00	0.53	0.13 to 0.93	0.43	0.17 to 0.69	0.43	0.16 to 0.70
**Age**	-0.27	-0.29 to -0.25	-0.28	-0.30 to -0.25	-0.12	-0.14 to -0.11	-0.13	-0.14 to -0.11
**Current smokers**			-1.17	-1.45 to -0.88			-0.82	-1.18 to -0.46
**Drinkers**			0.49	0.32 to 0.67			0.19	0.05 to 0.34
**Low physical activity**			0.12	0.02 to 0.21			0.09	0.02 to 0.16
**Small number of meals (<3 times)**			0.25	0.02 to 0.49			-0.41	-0.59 to -0.23
**Poor dietary intake**			-0.03	-0.12 to 0.06			-0.05	-0.12 to 0.02
**Severe economic status**			-0.02	-0.12 to 0.08			-0.02	-0.09 to 0.05
**Psychological distress**			-0.07	-0.18 to 0.04			-0.10	-0.17 to -0.03
**Insominia**			-0.02	-0.15 to 0.10			-0.06	-0.14 to 0.01
**Low level of social network**			-0.04	-0.14 to 0.05			0.01	-0.05 to 0.08
**Low level of social capital**			0.04	-0.09 to 0.17			-0.02	-0.12 to 0.08

CI, confidence interval; TH, temporary housing group.

The number of participants represents the individuals who participated in at least one survey from 2011 to 2015 and had complete data of all variables used in each model in one’s participated survey out of the original 9,909 participants.

Model 1: adjustment for age, time, living conditions, the interaction between living conditions and time points.

Model 2: model 1 plus smoking status, alcohol drinking status, physical activity, the number of meals (<3 times), dietary intake, economic status, psychological distress, insomnia, social network and social capital.

Coefficients and 95% confidence intervals were calculated using the linear mixed effect models.

eTable 3. Coefficient of body weight in mixed effects using linear mixed effects models with the missing covariate data in the 2011 survey by multiple imputation methods

	Men				Women			
	Model 1 (n=3,933)		Model 2 (n=3,933)		Model 1 (n=6,147)		Model 2 (n=6,147)	
	Coefficient (kg)	95% CI	Coefficient (kg)	95% CI	Coefficient (kg)	95% CI	Coefficient (kg)	95% CI
**TH**	-0.18	-0.47 to 0.11	-0.12	-0.41 to 0.17	-0.30	-0.51 to -0.10	-0.29	-0.50 to -0.09
**2011**	Base		Base		Base		Base	
**2012**	-0.02	-0.13 to 0.08	0.02	-0.09 to 0.13	0.15	0.08 to 0.23	0.18	0.10 to 0.26
**2013**	0.13	-0.02 to 0.28	0.15	0.00 to 0.31	0.16	0.06 to 0.26	0.19	0.08 to 0.30
**2014**	0.29	0.10 to 0.47	0.36	0.17 to 0.55	0.20	0.07 to 0.32	0.24	0.11 to 0.37
**2015**	0.45	0.23 to 0.66	0.54	0.32 to 0.77	0.19	0.05 to 0.34	0.25	0.09 to 0.41
**2012 × TH**	0.46	0.28 to 0.64	0.43	0.25 to 0.62	0.48	0.36 to 0.61	0.47	0.34 to 0.60
**2013 × TH**	0.54	0.29 to 0.80	0.57	0.31 to 0.83	0.61	0.44 to 0.79	0.63	0.45 to 0.81
**2014 × TH**	0.51	0.19 to 0.82	0.44	0.12 to 0.76	0.69	0.48 to 0.90	0.69	0.47 to 0.91
**2015 × TH**	0.62	0.24 to 1.00	0.54	0.15 to 0.93	0.44	0.18 to 0.69	0.46	0.19 to 0.72
**Age**	-0.26	-0.27 to -0.25	-0.27	-0.28 to -0.25	-0.12	-0.13 to -0.11	-0.12	-0.13 to -0.12
**Current smokers**			-1.13	-1.27 to -0.98			-0.85	-1.20 to -0.50
**Drinkers**			0.53	0.44 to 0.61			0.20	0.09 to 0.31
**Low physical activity**			0.12	0.03 to 0.21			0.08	0.02 to 0.15
**Small number of meals (<3 times)**			0.23	0.00 to 0.46			-0.35	-0.52 to -0.17
**Poor dietary intake**			-0.04	-0.13 to 0.05			-0.06	-0.13 to 0.00
**Severe economic status**			-0.02	-0.12 to 0.08			-0.02	-0.09 to 0.05
**Psychological distress**			-0.06	-0.17 to 0.05			-0.09	-0.16 to -0.02
**Insominia**			-0.03	-0.16 to 0.09			-0.08	-0.15 to 0.00
**Low level of social network**			-0.04	-0.13 to 0.06			0.01	-0.05 to 0.08

**Low level of social capital**	0.04	-0.09 to 0.17	-0.03	-0.13 to 0.06

CI, confidence interval; TH, temporary housing group.

Model 1: adjustment for age, time, living conditions, the interaction between living conditions and time points.

Model 2: model 1 plus smoking status, alcohol drinking status, physical activity, the number of meals (<3 times), dietary intake, economic status, psychological distress, insomnia, social network and social capital.

Coefficients and 95% confidence were calculated using the linear mixed effect models.

eTable 4. Pairwise comparison of the difference of body weight in living conditions using linear mixed effect models

	**Men**				**Women**			
	**Model 1 (n=3,852)**		**Model 2 (n=3,818)**		**Model 1 (n=6,057)**		**Model 2 (n=6,004)**	
	**Mean difference (kg)**	**95% CI**	**Mean difference (kg)**	**95% CI**	**Mean difference (kg)**	**95% CI**	**Mean difference (kg)**	**95% CI**
**2011**	-0.19	-0.48 to 0.10	-0.12	-0.42 to 0.18	-0.31	-0.51 to -0.10	-0.28	-0.49 to -0.07
**2012**	0.28	0.02 to 0.54	0.31	0.04 to 0.58	0.18	0.00 to 0.36	0.17	-0.02 to 0.35
**2013**	0.37	0.13 to 0.62	0.46	0.20 to 0.72	0.31	0.14 to 0.48	0.33	0.15 to 0.51
**2014**	0.32	0.05 to 0.59	0.32	0.03 to 0.60	0.39	0.20 to 0.57	0.39	0.20 to 0.58
**2015**	0.43	0.08 to 0.77	0.41	0.05 to 0.77	0.12	-0.11 to 0.36	0.15	-0.09 to 0.39

CI, confidence interval; HR, hazard ratio.

Reference= not temporary housing group.

Model 1: adjustment for age, time, living conditions, the interaction between living conditions and time points.

Model 2: model 1 plus smoking status, alcohol drinking status, physical activity, the number of meals (<3 times), dietary intake, economic status, psychological distress, insomnia, social network and social capital.

Coefficients and 95% confidence intervals were calculated using the linear mixed effect models.

eTable 5. The coefficients for body weight between time and living conditions stratified by obesity in the 2011 survey using multivariate linear mixed models

		Men				Women			
		Obesity (n=1,446)		No obesity (n=2,406)		Obesity (n=1,792)		No obesity (n=4,253)	
		Coefficient (kg)	95% CI	Coefficient (kg)	95% CI	Coefficient (kg)	95% CI	Coefficient (kg)	95% CI
**Obesity**	**2012 × TH**	0.51	0.16 to 0.86	0.41	0.21 to 0.62	0.61	0.35 to 0.87	0.43	0.29 to 0.57
	**2013 ×TH**	0.62	0.13 to 1.10	0.48	0.20 to 0.77	0.76	0.39 to 1.13	0.56	0.37 to 0.75
	**2014 ×TH**	0.62	0.02 to 1.22	0.39	0.05 to 0.74	0.89	0.44 to 1.34	0.61	0.38 to 0.84
	**2015 ×TH**	0.74	0.01 to 1.47	0.52	0.09 to 0.94	0.71	0.17 to 1.25	0.33	0.05 to 0.61

CI, confidence interval; TH, temporary housing group.

Obesity was identified as BMI of ≥25 kg/m^2.

Coefficients and 95% confidence were calculated using the linear mixed effect models adjusted for age, time, living conditions, the interaction between living conditions and time points.

eTable 6. Coefficient of mixed effects with the interaction in time, living conditions, and obesity in the 2011 survey using linear mixed effects models

	Men (n=3,952)			Women (n=6,055)		
	**Coefficient (kg)**	**95% CI**	***P* Value**	**Coefficient (kg)**	**95% CI**	***P* Value**
**2012 × TH × obesity**	0.22	-0.39 to 0.83	0.479	0.22	-0.21 to 0.65	0.324
**2013 × TH × obesity**	0.02	-0.61 to 0.65	0.954	0.08	-0.36 to 0.53	0.710
**2014 × TH × obesity**	0.02	-0.66 to 0.70	0.963	0.06	-0.42 to 0.54	0.803
**2015 × TH × obesity**	-0.13	-0.90 to 0.63	0.732	-0.30	-0.85 to 0.24	0.270

CI, confidence interval; TH, temporary housing group.

Obesity was identified as BMI of ≥25 kg/m^2.

Coefficients and 95% confidence intervals were calculated using the linear mixed effect models adjusted for age, time, living conditions, the interaction among living conditions, obesity, and time points.

eTable 7. Detailed characteristics of the participants in the 2011 survey (n=9,909)

		Men (n=3,852)					Women (n=6,057)				
		Missing	TH (n=1,231)	non-TH (n=2,621)			Missing	TH (n=1,938)	non-TH (n=4,119)		
		n (%)	Mean (SD)	Mean (SD)	*P* Value		n (%)	Mean (SD)	Mean (SD)	*P* Value	
**Physical activity**	**Physical activity**	18 (0.00)	12.0 (2.8)	12.3 (2.7)	0.001	*	43 (0.01)	11.8 (2.6)	12.1 (2.5)	<0.001	*
**Number of intakes per day**	**Staples foods (rice, bread, and noodles)**	10 (0.00)	2.8 (0.5)	2.8 (0.5)	0.061		8 (0.00)	2.8 (0.5)	2.9 (0.4)	0.067	
	**Meat**	100 (0.03)	0.9 (0.7)	0.9 (0.7)	0.097		140 (0.02)	0.9 (0.6)	0.9 (0.6)	0.447	
	**Fish and shellfish**	27 (0.01)	1.3 (0.7)	1.4 (0.7)	<0.001	*	45 (0.01)	1.25 (0.67)	1.30 (0.68)	0.007	*
	**Egg**	91 (0.02)	1.0 (0.7)	1.1 (0.7)	0.203		108 (0.02)	1.0 (0.6)	1.0 (0.6)	0.927	
	**Soybean and product (tofu and fermented soybeans)**	41 (0.01)	1.4 (0.8)	1.5 (0.8)	<0.001	*	43 (0.01)	1.4 (0.8)	1.5 (0.8)	<0.001	*
	**Vegetables**	22 (0.01)	2.0 (0.9)	2.2 (0.9)	<0.001	*	31 (0.01)	2.3 (0.8)	2.4 (0.8)	<0.001	*
	**Fruit**	61 (0.02)	1.2 (0.9)	1.3 (0.9)	<0.001	*	66 (0.01)	1.5 (0.9)	1.6 (0.9)	0.162	
	**Dairy products (milk, yogurt, and cheese)**	49 (0.01)	1.0 (0.8)	1.1 (0.8)	0.126		39 (0.01)	1.2 (0.8)	1.2 (0.8)	0.807	
**Psychological distress**	**K6 scale**	36 (0.01)	4.3 (4.4)	3.7 (4.2)	<0.001	*	123 (0.02)	5.7 (4.8)	4.9 (4.6)	<0.001	*
**Insomnia**	**Athens Insomnia Scale**	39 (0.01)	0.7 (0.5)	0.8 (0.4)	<0.001	*	99 (0.02)	0.6 (0.5)	0.7 (0.5)	<0.001	*
**Social network**	**Lubben Social Network Scale**	75 (0.02)	13.0 (6.6)	13.1 (6.7)	0.698		127 (0.02)	13.1 (5.9)	13.2 (5.9)	0.585	
**Social capital**	**Social cohesion**	8 (0.00)	11.5 (2.7)	11.8 (2.9)	0.006	*	23 (0.00)	11.8 (2.7)	12.1 (2.8)	<0.001	*

TH, temporary housing group; SD, standard deviation

With regard to physical activity, total point scores range from 3 to 15. In Social cohesion, total point scores range from 0 to 16.

Variables indicate the mean (standard deviation). Means and standard deviations were calculated using Student's t-tests.

*Statistically significant (P<0.05).
